# Effects of microtubule-inhibiting small molecule and antibody-drug conjugate treatment on differentially-sized A431 squamous carcinoma spheroids

**DOI:** 10.1038/s41598-020-57789-y

**Published:** 2020-01-22

**Authors:** Kenneth R. Durbin, M. Shannon Nottoli, Gary J. Jenkins

**Affiliations:** Drug Metabolism and Pharmacokinetics, AbbVie, Inc, North Chicago, IL 60064 United States

**Keywords:** Cancer models, Chemotherapy, Molecularly targeted therapy, Drug development, Preclinical research

## Abstract

Multicellular tumor spheroids have been increasingly used by researchers to produce more physiologically relevant experimental environments. However, tracking of spheroid growth and treatment-induced volume reduction has not been readily adopted. Here, squamous carcinoma cells were seeded at different starting cell numbers with growth and reduction kinetics monitored using live cell imaging. Following the initial growth phase, spheroids were treated with auristatin as small molecule (MMAE) or as antibody-drug conjugate containing non-cleavable auristatin drug payload (033-F). Compared to cells in monolayers, 033-F had notably weaker potency against spheroids despite potency levels of MMAE being similar against monolayers and spheroids. Accumulation of released payload from 033-F was reduced in higher volume spheroids, likely contributing to the potency differences. Despite lowered potency towards spheroids with 033-F, spheroid volume was still readily reduced by 033-F in a dose-dependent fashion, with >85% volume reductions at the highest concentrations for all spheroid sizes. Additionally, the core of the larger spheroids showed more resiliency towards microtubule inhibition. Overall, this work highlights how various *in-vivo* ‘features’ such as tumor penetration, cell interactions, and increased resistance to therapeutics can be integrated into a spheroid model and tracked over time by automated imaging technology.

## Introduction

In their native space, tumor cells experience interactions with neighboring cells over their entire surface area. This environment is vastly different than the conditions used for *in vitro* studies where cells attach to plastic dishes and grow in two-dimensions (2D) as monolayers. While many crucial molecular and cellular biology phenomena have been elucidated using cells cultivated on 2D surfaces, cell-to-cell communications, spatial orientation in the tissue, cell interactions with the surrounding matrix, and physiological signaling cannot be properly recreated^1^. Growing cells into three-dimensional (3D) spheroids can better capture aspects of tumor biology such as regions of high oxygen, nutrients, and subsequently proliferation, as well as regions of low nutrients and hypoxia that can lead to cell quiescence and ultimately necrosis^2^. A wide number of options for generating 3D spheroids has been put forth in the literature, including hanging drop, matrix-based methods, spinner flasks, and ultra-low adhesion plates, with each having its particular advantages and disadvantages^3^.

Due to approval rates under 10% for new therapeutics in oncology, improved methods of screening are needed to find better drugs to bring into clinical studies^4^. More predictive models of drug efficacy and toxicity would help filter out candidates with a low chance of clinical success. Currently, many of the early-stage drug screens are performed with traditional 2D monolayers. Preclinical validation experiments typically lack many characteristics of the natural tumor milieu, instead presenting an environment that can be a stark departure from an *in vivo* tumor. Previous and current 2D screening paradigms may introduce significant biases during the initial selection stages of early drug discovery ‘hit-to-lead’ cellular screening approaches^5^, potentially advancing drug molecules with limited potential or even excluding promising drug molecules from moving further forward in drug development. Spheroids on the other hand have been successfully used to better recreate the gene expression patterns of *in vivo* tissues^6,7^ as well as predict *in vivo* tumor sensitivity to drug previously missed by 2D screens^8^.

Different types of therapeutics pose additional new challenges. Protein therapeutics are significantly larger than small molecules in both molecular weight and physical radius. The ability to more closely replicate the natural physiological environment during screening may allow researchers to better understand key differences in how well a biologic can penetrate a tumor. Such considerations become especially pronounced for more complicated solid tumor therapeutics. For example, antibody-drug conjugates (ADCs) combine elements of receptor targeting via a specific antibody with small molecule inhibition in an effort to mediate greater efficacy than antibody or small molecule inhibition alone^9^. Antibody therapeutics typically only need to bind extracellular antigen to elicit an anti-cancer effect, whereas the majority of ADCs need to be brought into the cell in order to release the active drug payload. Having a preclinical evaluation system in place that incorporates aspects of tumor penetration, realistic antigen behavior, and small molecule-mediated cell killing over a relevant time period is important to fully evaluate whether the ADC is a viable drug candidate. For example, the number of antigens present on the cell surface becomes instrumental in dictating possible efficacy; levels of the frequently targeted antigens used in a number of ADCs (e.g., ErbB family members EGFR and HER2) have been shown to change between 2D and 3D models^10^. Furthermore, certain spheroids can also have changed internalization biology, producing significantly altered rates of intracellular drug accumulation^11^.

While 3D platforms offer demonstrable benefits over standard 2D-based cell culture methods, the use of 3D technologies in drug discovery and screening efforts has been minimal, often due to throughput^12^ or efficacy determination challenges^13^. Because of the massive scale of many industrial drug development activities, reproducibility, cost, and ease of setup are paramount in the successful rollout of 3D technologies^14^. Successes have therefore focused on high-throughput methods providing both ease-of-use and compatibility with automation, which to-date has meant using ultra-low adhesion plates for spheroids formation and imaging systems or viability measurements for drug potency determination^15,16^. However, the current mainstream techniques do not capture time-based dynamics of spheroid size, missing valuable information regarding the rate and completeness of spheroid regression^17^. Limited data is also available regarding the influence of spheroid size on overall drug potency. Here, a high-content approach using low adhesion culture plates and live cell imaging technology was used to characterize the sensitivity of squamous carcinoma cell spheroids to ADCs and small molecules at varying plating densities. This proof-of-concept approach represents the first high-content analysis of spheroid volume over time following ADC treatment and sets the stage for more advanced future preclinical assessments

## Results

### Spheroid growth

Tumor cells grown in close proximity on ultra-low adhesion material tend to form cell-to-cell contacts; in comparison, cells grown on culture-treated plates alternatively promote cell-to-plate associations over cell-to-cell interactions. Because the cell-to-cell contacts more closely resemble the interactions of cells found in their physiological tissue architecture, many types of cells will self-assemble into spheroids^18^. Here, A431 human squamous carcinoma cells expressing a fluorescent red nuclear protein were grown on round bottom ultra-low adhesion plates. The plated cells quickly came together into a spheroid, with a well-formed spherical shape observable in less than 12 hr (Supplemental Fig. 1 and Supplemental Videos 1 and 2).

**Figure 1:**
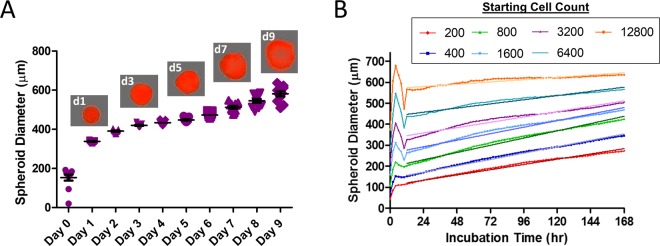
Spheroid growth kinetics. (**A**) A431 cells were seeded at 3200 cells in a round bottom ultra-low adhesion plate. Cell area for 11 spheroids was tracked over nine days and then converted to spheroid diameter. Images are from the same spheroid over the time course. (**B**) The number of starting cells plated was varied between 200 and 12800 cells and spheroid diameter was followed over seven days. Linear regressions were fit through the diameter growth data for each spheroid size. Data points are the mean values from experimental replicates comprised of the mean value of ≥11 spheroids per experiment.

A431 spheroids were tracked over 9 days using live cell imaging to measure the area of fluorescently labeled protein, which was then converted into a spheroid diameter (Supplemental Fig. 2). Consistent formation and growth kinetics were observed for spheroids originally seeded with 3200 cells per well and followed over a 9-day time frame (Fig. 1A). The spheroids had an average diameter (with standard deviation) of 338 µm (±5.5 µm) after 1 day and 581 µm (±42 µm) after 9 days with coefficients of variation of 1.6% and 7.2%, respectively. Steady and reproducible growth over 168 hr was also observed for spheroids where the starting cell counts was varied by powers of two between 200 and 12800 cells (Fig. 1B and Supplemental Fig. 3); however, when initial cell counts were extended to 25600 cells, inconsistent growth was observed (Supplemental Fig. 3B). For cells seeded at ≤12800 cells, spheroid diameter values generally stabilized 6–12 hr post-plating, which corresponded to the length of time needed for that number of cells to form a spheroid. Following stabilization, all spheroids plated at ≤12800 cells grew in diameter over the remainder of the time course. Diameters for the different spheroid seedings ranged from 119 µm to 741 µm after 12 hr and 323 µm to 683 µm after 168 hr (Fig. 1B and Supplemental Fig. 3). Following stabilization, growth rates were calculated by fitting the growth curve data to linear regressions (Fig. 1B). The average diameter growth rates were between 0.45 µm per hr and 1.43 µm per hr (Table 1). The smaller spheroids had greater overall percentage diameter and volume gains over the time course compared to the spheroids starting from a larger initial size (Supplemental Fig. 3D and Table 1). The spheroids seeded at 200 cells had the smallest spheroid volume after 7 days at 1.1 × 10^7^ µm^3^ while the spheroids seeded at 12800 cells had the largest volume of 1.4 × 10^8^ µm^3^ after 7 days (Supplemental Fig. 3E).

**Table 1:** Spheroid growth. Data shown are the mean values +/− SD. 7 d is calculated from the time that stable spheroids were achieved, which here was 12 hr post-plating.

Starting Cells	7 d Diameter (µm)	Diameter Growth (µm/hr)	7 d Diameter Change	7 d Volume Change
200	272.7 ± 35.6	1.04 ± 0.03	110 ± 51%	949 ± 597%
400	345.6 ± 20.8	1.26 ± 0.03	106 ± 30%	816 ± 375%
800	424.5 ± 55.8	1.43 ± 0.06	92 ± 32%	648 ± 337%
1600	467.9 ± 44.0	1.30 ± 0.07	68 ± 26%	402 ± 213%
3200	503.3 ± 26.0	1.06 ± 0.03	46 ± 11%	212 ± 73%
6400	565.9 ± 22.2	0.83 ± 0.04	27 ± 7%	105 ± 32%
12800	635.5 ± 10.2	0.45 ± 0.05	12 ± 4%	40 ± 14%

### Spheroid treatment

A431 squamous carcinoma cells have been shown to express high levels (i.e., ~1 × 10^6^ molecules per cell) of epidermal growth factor receptor (EGFR) on the cell surface^19,20^. Our in-house produced version of cetuximab, Ab033 (same sequence but slightly different glycosylation pattern than cetuximab), was used to target EGFR^21,22^. Although cetuximab and Ab033 are cytotoxic to A431 cells as monoclonal antibodies^23,24^, Ab033 was additionally armed here with a non-cleavable payload featuring mono-methyl auristatin F attached to the reduced cysteines of Ab033 via maleimidocaproyl at an average drug-to-antibody ratio (DAR) of 4 (referred to here as 033-F). The intent of adding auristatin molecules to the 033 antibody was to investigate the platform as a potential system for assessing ADCs *in vitro* as well as further promote cell death through inhibition of microtubules by the cysteine-maleimidocaproyl-mono-methyl auristatin F (cys-F) payload that is released from 033-F following lysosomal degradation^25^. The non-cleavable, low permeability cys-F payload was selected to ensure any cell killing or spheroid reduction stemming from microtubule inhibition was a result of internalization and payload release within the cells and not bystander effect. Overall, 033-F has multiple mechanisms (i.e., EGFR inhibition via antibody binding and microtubule inhibition via drug binding) that can result in cell killing, with these mechanisms perhaps even working synergistically.

**Figure 2:**
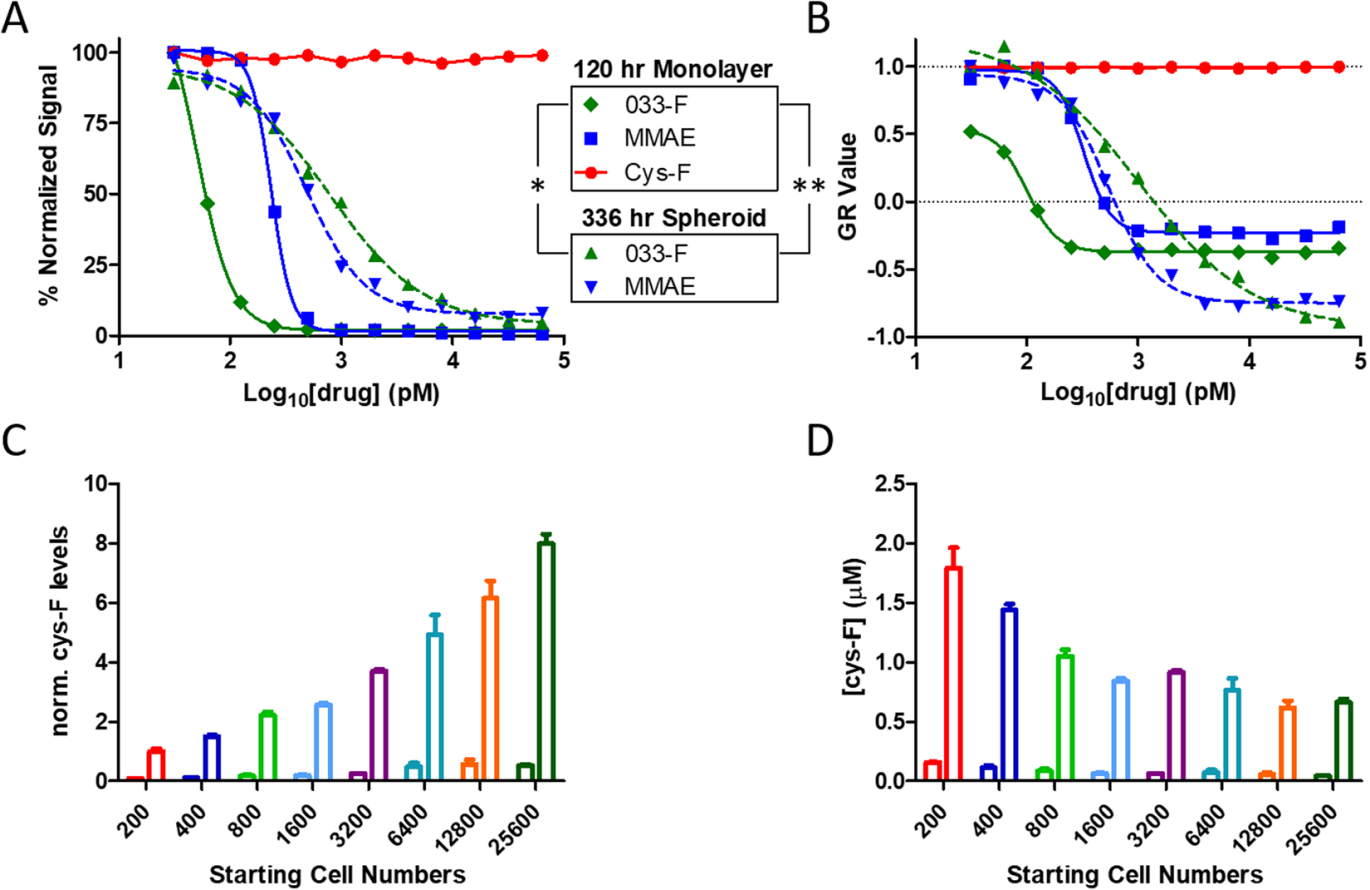
Treatment with microtubule inhibitors. (**A**) Monolayers and spheroids originating from 800 plated cells were treated for 120 hr or 336 hr, respectively, with serial dilutions of MMAE small molecule, cys-F, or 033-F ADC. Signal was normalized to the highest signal value from transfected nuclear proteins in each experiment obtained by live cell imaging. (**B**) Growth reduction (GR) values and curve fits were calculated from the same data set. P values less than 0.05 and 0.01 are denoted by * and **, respectively. (**C**) The levels of released drug payload, cys-F, in spheroids were analyzed by LC-MS after 4 hr and 24 hr incubations with 64 nM 033-F. Signals were normalized to the signal from the 200 cell spheroids at 24 hr. Values on the left of each grouping are 4 hr time points and values on the right are 24 hr time points. Error bars are standard deviation. (**D**) The concentration of drug payload was then calculated for the spheroids by normalizing each signal by the spheroid volume.

To obtain a benchmark of A431 cell susceptibility to microtubule inhibitors (MTI), A431 monolayers were treated with either an ADC, a drug linker, or a small molecule: the forms used were the 033-F ADC, the low permeability 033-F release product cys-F (which is also the full drug linker in this case because of the non-cleavable linker), or the permeable auristatin drug variant mono-methyl auristatin E (MMAE)^26^, respectively. The total signal from fluorescent protein expressing A431 cells was readily decreased by MMAE and 033-F, indicating cell killing. Following 120 hr of treatment, MMAE and 033-F yielded IC50 drug potency values of 236 pM and 51 pM, respectively (Fig. 2A). Conversely, treatment with the low permeability cys-F drug-linker was unable to produce an effect at any of the concentrations used and thus was not used in any additional experiments throughout this study.

To compare potency with the spheroid format, A431 cells were next seeded at a middle range of cell concentrations, with 800 cells plated per well and allowed to form spheroids and grown for 9 days. The matured spheroids were then treated with 033-F and MMAE for 14 days. Dose-proportional responses were observed for both treatment types (Fig. 2A). IC50 values of total cellular red signal after 336 hr were 493 and 791 pM for MMAE and 033-F, respectively. The differences between 2D and 3D 033-F potency values were statistically significant (p-value < 0.05) while the values between 2D and 3D MMAE, as well as 3D MMAE and 3D 033-F were not.

Previous literature has demonstrated how different growth rates can impact metrics regarding drug sensitivity and potency^27^. As spheroids and monolayers often have substantially different growth rates^28^, additional analyses were needed to determine if growth rate differences were influencing cellular potency IC50 values. By using the normalized growth rate inhibition (GR) values that have been introduced by Sorger’s group over the last few years^27–29^, the influence of division rates on potency metrics can be minimized^29^. GR values were therefore calculated for both spheroid and monolayer data (Fig. 2B). The MMAE GR50 values were nearly identical, with a spheroid GR50 of 320 pM MMAE and monolayer GR50 of 291 pM MMAE. Conversely, calculated GR50 and IC50 values for 033-F treated spheroids and monolayers had significant differences in observed potency values between monolayers and spheroids (a p-value < 0.01 for GR50 values and a p-value < 0.05 for IC50 values).

To further investigate the differences in 033-F monolayer and spheroid potency values, spheroids were incubated with the same high concentration of 033-F and the concentration of released intracellular cys-F drug was evaluated at various time points via mass spectrometry analysis e (Fig. 2C). The signal of cys-F from spheroids after 1 hr of incubation with 033-F was below the limit of quantitation and was therefore not included. However, after 4 hr with 033-F, the cys-F levels were sufficiently high to be quantitated in all spheroid sizes. The total cys-F levels increased by 24 hr of incubation with 033-F, by 12.4-fold between 4 hr and 24 hr. For every two-fold increase in initial cell seeding number, the cys-F concentration was increased by an average of 32% and 35% at 4 and 24 hr, respectively. The raw values were converted to spheroid concentrations using volumes converted from imaging measurements (Fig. 2D). The spheroids plated at 200 cells had cys-F concentrations of 1.8 µM at 24 hr, which was the highest of all the different spheroid sizes and represented a nearly 20-fold increase compared to the media concentration of ADC.

**Figure 3:**
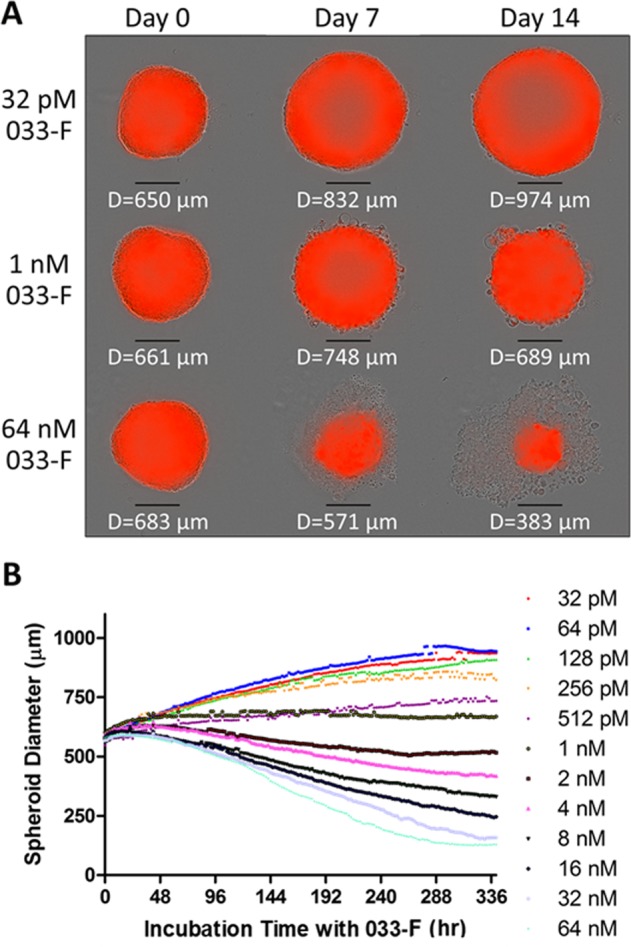
Spheroid treatment with 033-F ADC. (**A**) Spheroids were originally plated at 800 cells and allowed to grow for 9 days prior to treatment with 033-F. Day 0 indicates when treatment was started following the initial 9-day growth period. Images are shown for spheroids treated with the low, medium, and high concentrations of 033-F. Scale bars beneath each spheroid image are 300 µm in length. (**B**) Spheroid growth and reduction were tracked over 14 days while in the presence of 033-F. Data points are mean hourly values from three biological replicates.

At the individual spheroid level, spheroids originally plated at 800 cells per well and treated with 64 nM 033-F saw a reduction in spheroid size that occurred gradually over the time course. Cell death proceeded from the periphery of the spheroid inwards (Fig. 3A, Supplemental Video 3). Decreases to spheroid size were observed for the highest concentrations of 033-F and MMAE (>1 nM and up to 64 nM for both) over the full treatment period while growth progressed for the spheroids treated at the lowest concentrations (Fig. 3B, Supplemental Fig. 4, and Supplemental Videos 3–5). Spheroid volume was stabilized at ~1 nM for both 033-F and MMAE. After 336 hr in the presence of the highest 033-F concentration that was used (64 nM 033-F), the diameter and volume of the spheroids were demonstrably reduced, having decreases from pre-treatment starting values by up to 78% and 94%, respectively (Supplemental Fig. 5, left). Spheroids treated with this highest concentration had diameters that were 86% smaller and volumes that were reduced by 98% after 336 hr compared to the spheroids subjected to the lowest tested 033-F concentration. For MMAE treatment, the spheroids incubated with the highest concentration of MMAE had diameters that were 71% lower and volumes were decreased by 97% versus those spheroids treated with the lowest MMAE concentration (Supplemental Fig. 5, right).

**Figure 4:**
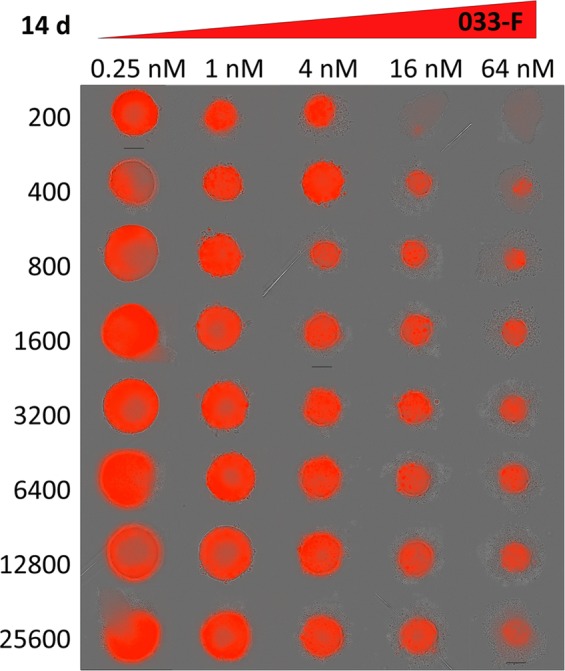
Spheroid Reductions with 033-F. Representative images of differentially-sized spheroids from the same experiment after 14 d of 033-F treatment are shown. Scale bars are 300 µm.

**Figure 5:**
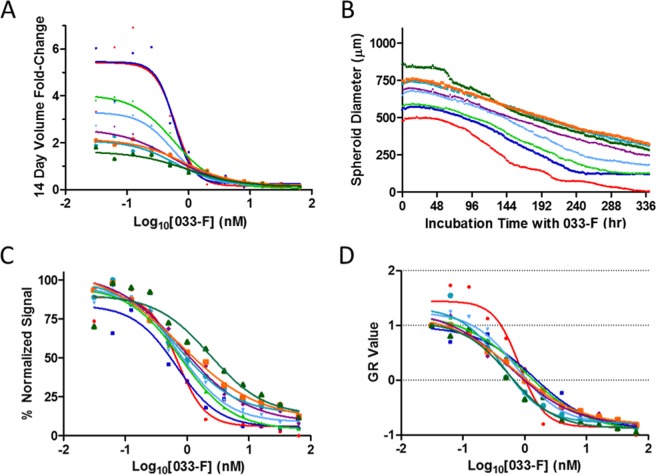
033-F treatment of different spheroid sizes. Spheroids were plated at varying initial concentrations of cells and grown for 9 days. After the initial growth phase, the spheroids were treated with 033-F over 14 days. (**A**) Volume changes were monitored over the time course and fold-changes were reported using 14-day volumes compared to the volumes prior to treatment. (**B**) The diameter of spheroids treated with 64 nM 033-F were tracked using live cell imaging over 14 days. (**C**) Total area of each spheroid group after 14 days was normalized to the highest values from each treatment concentration. IC50 curves were fit to the data. (**D**) Total area values after 14 days were also converted into GR values and then IC50 curves were fit to the data. All values are mean values from three experimental replicates.

Next, the size of spheroids was varied to better understand how MMAE and 033-F treatment would be impacted by changing tumor size. As above, A431 cells were grown into spheroids for 9 days after being plated at a seeding density between 200 and 25600 cells. Spheroids were then treated with 033-F for 14 days (Fig. 4 and Supplemental Fig. 6). Similar to spheroids dosed with 033-F described above, substantial size reductions were observed at high concentrations of 033-F while growth still occurred when lower concentrations were used (Fig. 5A and Supplemental Fig. 7). Of note, all spheroid sizes treated at the lowest concentrations except the 200-cell batch reached a similar size plateau around 900 µm in diameter. At the highest 033-F doses, volumes for every size of spheroid were reduced from their starting values by at least 85% after 336 hr of treatment (Fig. 5A,B). The spheroids given the lowest 033-F concentration doses all had increased volume, with the largest spheroids nearly doubling in volume and the smallest spheroids growing even more substantially (volume increases of >500%).

033-F IC50 values were calculated after 14 days in the presence of drug; the IC50 values were within three-fold across all spheroid sizes (Fig. 5C). The average IC50 values for the spheroids slightly increased for larger sized spheroids, from a low of 0.67 nM for the 400-cell spheroids to a high of 1.76 nM for the 25600-cell spheroids (Table 2). In addition, the Hill coefficients for the best IC50 curve fits decreased as tumor size increased. Lower Hill coefficients indicate a more gradual reduction in spheroid size as a function of increasing drug concentration. Because spheroid volume increases at the lowest treatment concentration varied from nearly 6-fold for spheroids on the smaller end of the size spectrum to 0.5-fold for spheroids at the high end of the size spectrum (Table 2), we wanted to assess if spheroid growth was factoring into the reported IC50 values. GR50 values were therefore calculated for all the different size spheroids (Fig. 5D), and indeed, after normalizing for spheroid growth, the GR50 values trended down as the spheroids increased in size, from 0.81 nM for the small spheroids to 0.27 nM for the larger spheroids (Table 2). Emax values are also included in Table 2 for comparison of the maximum spheroid reduction that can be achieved for the various spheroid sizes.

**Table 2:** Spheroid treatment metrics over 7 d.

Starting Cells	0 hr Diameter (µm)	IC50 (nM)	GR50 (nM)	Emax	Max. Vol. Decrease	Max. Vol. Increase
200	466.0 ± 103	0.98	0.81	1 × 10^−4^	100%	590 ± 213%
400	522.3 ± 78	0.67	0.47	0.043	93 ± 7%	507 ± 169%
800	591.4 ± 69	1.00	0.50	0.052	94 ± 6%	296 ± 35%
1600	637.7 ± 53	1.19	0.51	0.079	92 ± 8%	268 ± 76%
3200	685.6 ± 58	1.38	0.35	0.092	91 ± 8%	169 ± 51%
6400	722.6 ± 43	1.34	0.33	0.13	88 ± 9%	128 ± 34%
12800	749.6 ± 73	1.52	0.35	0.14	86 ± 12%	109 ± 62%
25600	801.4 ± 74	1.76	0.27	0.17	87 ± 12%	50 ± 19%

## Discussion

For traditional drug screening paradigms using 2D cell culture methodology, positive and negative results may arise from biased experimental setups. Instead of being ‘true’ hits, these results may instead be comprised of false positives and false negatives. To reduce these false readouts, 3D cell culture and its closer representation of the *in vivo* environment is increasingly being looked to for better prediction of which drug candidates will be successful in the future. However, to get 3D systems into drug discovery workflows, current automated and well-honed 2D high-content screening assays will need to be transitioned to an amenable 3D alternative. Here, straightforward imaging techniques were implemented to monitor spheroid size over time in the presence of drug. One of the potential issues with imaging is the accurate measurement of spheroid size, especially in a high-content assay where many objects must be measured across many time points. By transfecting cells with a fluorescent nuclear protein, a more accurate detection of spheroid diameter could be implemented. Accurate measurement of spheroid diameter was further enabled by setting the fluorescence threshold to best remove background signal, single cells around the spheroid, and dying or dead portions of cells at the periphery of the spheroid. Using a high signal threshold led to significantly better representations of spheroid size compared to masking of phase contrast images, which led to inclusion of cellular debris (Supplemental Fig. 2).

A key aspect for reliable drug discovery assays is reproducible results. In the case here, A431 cells readily formed spheroids at different starting cell concentrations ranging between 200 and 25600 cells. Following initial spheroid formation, all spheroids except the high cell concentration group showed reproducible growth that was consistent and steady. The spheroids in the high cell group exhibited limited growth, with some of the spheroids exhibiting spheroid diameter reductions over time (Supplemental Fig. 3B). The growth of the smaller spheroids was evident over an entire initial 168 hr growth period (Fig. 1B). Additionally, spheroid volume changes were highest for spheroids seeded at lower cell numbers, likely due to higher accessibility to nutrients (Table 1). Because the spheroids used in this work lacked vasculature, the delivery of nutrients and oxygen across the spheroids would become limited past a certain threshold, which here appears to be around 750 µm for spheroids plating at high density (Supplemental Fig. 3). Interestingly, the spheroids plated at a lower density were able to expand to larger sizes than those spheroids plated at high initial density. In particular, spheroids with lower original seeding numbers that were treated with the lowest concentration of 033-F were able to grow to a diameter of nearly 1000 µm by the end of the treatment period, exceeding the size of the spheroids originally plated at 25600 cells (Fig. 3 and Supplemental Fig. 7). Those spheroids plated at 25600 cells were unable to expand beyond their initial spheroid size. Prior observations of spheroids with diameters greater than 500–600 µm have shown a limiting phenomenon, as larger spheroids tend to form a hypoxic core composed of necrotic cells as well as a gradient of nutrients and gases that decreases towards the center of the spheroid (Friedrich 2007). Growing spheroids from a smaller starting size allows for a natural expansion of the spheroid, possibly leading to a more organic spheroid architecture being formed.

Because many of the conditions in the experiments performed for this work had different growth rates (e.g., 2D and 3D, small spheroids and large spheroids), traditional potency metrics may not be suitable for comparing the sensitivity of the different growth conditions to each other^29^. By utilizing a normalization factor for cell growth dynamics, the influence of growth rate on standard potency values such as IC50 can be minimized^29^. These normalized GR values should therefore also enable more robust comparisons of drug potency across different lines and experiments, which would be especially helpful for 2D and 3D comparisons of response to drug treatment. Spheroids are often cited as being more chemo-resistant than 2D monolayers^30^. However, the doubling rate of spheroids can be substantially slower than their 2D counterparts, leading to a potential for bias in the reported potency differences. To confirm if doubling times were impacting the potency values for 033-F and producing the 033-F sensitivity differences between 2D and spheroid models, GR values were calculated, and the results confirmed the potency differences were not an artifact of different doubling times (Fig. 2). When comparing the MMAE IC50 values for spheroid versus monolayer, a ratio of 2.1 was found between their IC50 values. In contrast, their GR50 values had a ratio of 1.1. The ratios of GR50 values for spheroids and monolayers were closer to one another, demonstrating influence of growth differences on the IC50 values. Similarly, GR values revealed the 033-F IC50 values of spheroids to be dependent on different growth kinetics between the differentially sized spheroids. The 033-F IC50 values in general trended up as the spheroid size increased. Once normalized to GR values, the sensitivity to 033-F was inversely correlated with size, becoming more sensitive to 033-F as size increased (Table 2).

While the 2D monolayer and 3D spheroid models showed similar sensitivity to the permeable small molecule MMAE, the IC50 and GR50 values for the ADC 033-F were significantly different (Fig. 2A,B). Likely explanations for the difference in sensitivity to 033-F could be either higher sensitivity to 033 antibody binding of EGFR in the 2D models compared to the 3D models or lowered penetration leading to less ADC binding of EGFR in spheroids. Previous work has demonstrated the existence of an ‘antigen barrier’ for antibodies and subsequently antibody-drug conjugates^31^. For antigen levels above a sufficient amount, antibody cannot permeate across the entire spheroid due to the high levels of antigen acting as a sink and is instead confined to the outer edges of the spheroid. Limited tumor penetration of both small molecule and ADCs is also in line with observations of spheroid cell killing by both 033-F and MMAE occurring at the periphery and then slowly working inwards over time (Supplemental Video 3). Indeed, previous work has also shown small molecule drugs to have lower penetration in larger spheroids; penetration of doxorubicin was more limited in larger spheroids and consequently the larger spheroids had higher IC50 values^32^. Additionally, the observation by mass spectrometry of larger spheroids having lower concentrations of released cys-F compared to smaller spheroids also corroborates lower tumor penetration for larger spheroids (Fig. 2D). Together the data indicates that although the GR50 values are lower for larger spheroids, which indicates effective killing of some portion of the spheroid (e.g., the periphery), the overall shape of the GR curves show a flatter curve for large spheroids because of the difficulty in killing cells closer to the center of the spheroid (Fig. 4D).

The high expression of EGFR in many cancer cells makes those cell types strong candidates for treatment with monoclonal antibodies and ADCs directed against EGFR. In particular for ADCs, the internalization pattern of EGFR when bound by an anti-EGFR antibody has led researchers to investigate different biologics that were initially dosed as monoclonal antibodies to instead be the backbone antibody in ADCs, including the clinical candidate ABT-414, as well as preclinical studies using cetuximab-containing ADCs^25,33^. The internalization and trafficking of EGFR antibodies to the lysosome has generally been shown to occur on the order of minutes; however, the release of cys-F from 033-F can lag by comparison^21^. The levels of cys-F were minimal after 1 hr of time with 033-F, registering below the limit of quantitation of the assay. In comparison, after 4 hr even the smallest spheroids contained ~50-fold higher levels than the limit of quantitation for the mass spectrometry measurements. In addition, the intracellular concentrations after 24 hr increased by more than 12-fold from the 4 hr time point. These large increases over time are significantly larger than the fold-increases observed in 2D cultures^16^. Time points were limited to 24 hr to prevent cell death from interfering with intracellular concentration measurements. Another observation was that when cys-F levels were converted to concentrations, the smaller spheroids had the highest 033-F concentrations. Previous literature has demonstrated a moving ‘antibody front’ that can take hours for spheroid penetration to occur and subsequent receptor binding to be established^34,35^. Such time frames are in stark contrast to 2D cultures that can be fully saturated by antibody within minutes. Because of this additional time needed to penetrate the spheroid, particularly for larger spheroids, the delivery of ADC can be delayed by several hours when compared to 2D cultures. Further, since full antibody catabolism is likely necessary for release of the drug payload from this non-cleavable ADC construct^36^, the slower release kinetics in combination with the time needed to penetrate the spheroid lends an explanation for the delay seen in the intracellular accumulation of cys-F. Despite the slower time frame of release, the low permeability of cys-F will allow the active payload to be retained in the cell over longer periods of time than a more permeable payload. Lastly, as mentioned above, an ‘antigen barrier’ may also be reached, especially for cells expressing high levels of receptor such as the A431 cells used in this study^31^.

Eight cell seeding levels were used to grow differentially sized spheroids, which were then treated with 033-F for two weeks. All tumor spheroids were diminished in the presence of 033-F or MMAE. The sensitivity to 033-F between the different spheroids was fairly comparable, with IC50 values spanning about two-fold and generally getting progressively higher as the spheroid size increased. However, the GR50 values were inverted after normalizing for growth rate and spanned three-fold in the opposite direction. While there were slight trend changes, sensitivity across the different spheroid sizes was similar, as all spheroids displayed marked chemo-resistance towards 033-F when compared to A431 cells cultured in monolayers. The volume reduction during treatment appeared to be from the outside-in, likely because the cells at the outside were exposed to a higher local concentration of drug than those further towards the core of the spheroids. One crucial observation was the ability to more completely diminish the interior of the spheroids as a function of initial spheroid size. The spheroids originating from a higher number of initial cells had cores that were the most resistant to both ADC and small molecule auristatin treatment, leading to less overall volume reductions and larger Emax values (Table 2). More explicitly, only the smallest spheroids were able to be completely eliminated with 033-F, whereas the largest spheroids were maximally reduced by 87% of their original volume. The combination of larger volume and longer time spent at such large volumes likely contributed to the spheroid core having limited access to oxygen and nutrients. As a consequence, the spheroid core may be less actively dividing than the cells at the perimeter of the spheroid as well as less actively dividing than the core cells of a smaller spheroid. The cells at the core may also be quiescent or necrotic. Mitotic cells are especially prone to microtubule inhibitors; consequently, microtubule inhibitors tend to be less cytotoxic to cells with lower rates of cellular division^37^. Further, the similar cell killing in terms of speed and outside-in manner for both MMAE and 033-F treatment points to decreased penetration of both. Small molecules and biologics have both been reported to have poor penetration in certain types of spheroids^32,38–40^.

With these results in mind, any of the spheroid sizes used here could provide useful data for drug discovery efforts in the future, although the end objective and type of drug modality being used should guide study design. For example, if antibodies with different binding affinity are being compared, a larger spheroid model may be more insightful due to tumor penetration potentially affecting efficacy outcomes *in vivo*. Also, large spheroids may provide a more heterogenous cell population, one comprised of a mixture of growing, quiescent, and necrotic cells, therefore bearing more resemblance to the mix of cells in a tumor that stem from the irregular vasculature. Such diversity could lead to insights regarding how a cancer responds to a chemotherapeutic agent at different physiological conditions. Alternatively, if different mechanisms of action are being tested or a shorter time course is desired, smaller spheroids may be preferred. For studies taking place with larger spheroids, it may be advantageous to grow the larger spheroids over several weeks instead of plating many cells at once. The spheroids given time to grow seemed to have less of a limitation on size and maintained growth throughout the time courses observed here. Measuring spheroid tumor densities in future studies could help normalize antibody and drug penetration levels for different cell lines. Using dye movement across spheroids over time could be used to define spheroid density. Overall, while it would be convenient to generalize these results to other cancer cells, different cell lines have a vast range of responses to being grown as 3D spheroids including sizes (e.g. compact versus loose formation) and shapes (e.g. round versus oblong), making such generalizations of limited utility^3^. For studies considering cells that produce spheroids that are not well-defined in shape (and many cell types do produce irregular shaped 3D tumors)^14^, the general workflow used here would still work; however, special and perhaps more elaborate mathematical considerations would need to be undertaken to properly define the geometry of the spheroid.

The work presented here showed that we were able to robustly and reproducibly grow spheroids in ultra-low adhesion plates and then perform long-term treatments of the spheroids with ADC and small molecule drug inhibitors. Through high-content imaging, large amounts of spheroid images could be taken over time and used to capture the spheroid volume kinetics. These data provide an informative look at the spheroid response to cytotoxic agents over time. Looking forward, this work could be readily expanded to high-throughput screening for potency in a variety of cancer cell models as well as included in preclinical safety testing on organoid models of normal tissues. To then further develop even more accurate representations of the natural cellular environment, co-cultures or organ-on-a-chip systems could be incorporated alongside live cell imaging^41,42^. Advancements in spheroid design have already been described in the literature, including angiogenesis models where endothelial networks are introduced into tumor spheroids to mimic the role that endothelial cells play in the tumor microenvironment^43^. Vascularization of the microtumors in an *in vitro* system has been demonstrated and could represent a major advance in the setup for *in vitro* experimentation^44^. Future studies could involve drug screening on intricately designed vascularized systems for more physiological relevance *in vitro* outcomes^45^. Lastly, great strides have been made in the areas of microfluidics^46^ and patient-derived spheroids^47^, so much so that one could envision future personalized clinical trials occurring in spheroids without the need for preclinical species^48^. In summary, the application of 3D cell culture and organs-on-a-chip for preclinical drug screening represents an exciting opportunity to accelerate the pace and success of drug discovery and development^49^.

## Materials and Methods

### Cells and spheroids

A431 cells were obtained from American Type Culture Collection. Cells and spheroids were grown in RPMI-1640 media supplemented with 10% fetal bovine serum. The cells were transfected with lentivirus to stably express mKate2 nuclear red fluorescent protein through the constitutive promoter EF-1α (Essen Bioscience, Ann Arbor, MI). Cells were at roughly 25% confluency at the time of infection. A multiplicity of infection of 3 was used and the cells were incubated with the lentivirus for 24 hr. The media was changed to fresh growth media and the cells were expanded for 24 hr prior to selection with puromycin. The high expression of EGFR by A431 cells was confirmed using flow cytometry^16^. Spheroids were formed by plating a defined number of cells onto clear round bottom ultra-low attachment 96 well plates (Corning) and centrifuging at 300 × g for 10 min. Spheroids were plated in the same manner regardless of initial starting cell numbers.

### Materials

Ab033 was produced as described previously^21^. 033-F was produced by incubating Ab033 in DPBS with two equivalents of tris(2-carboxyethyl)phosphine hydrochloride (TCEP) on ice for 5 hours and then incubating with cys-F at room temperature. Overall DAR was measured by hydrophobic interaction chromatography and confirmed to be an average of four.

### Live cell imaging

An Incucyte Zoom system (Essen Bioscience) was used for live cell imaging of cells and spheroids. Images were taken of each spheroid either every hour or every other hour. The round bottom plates allowed the newly formed spheroids to settle into a predictable location at the center of the well such that imaging could be accomplished by the Incucyte on a single image per well. The spherical shape that A431 cells take when grown as spheroids permitted diameters of the spheroid to be defined through straightforward geometric calculations. The total red area per image was used in calculating spheroid diameter by solving for diameter of a circle when the area of a circle is known. To obtain the total area of the red signal, a fixed threshold of 10 mean image fluorescence (RCU was used to differentiate spheroid from background). This value was higher than the typical signal a single cell would produce, which allowed for any single cells on the outer spheroid perimeter, as well as dead cells, to be excluded during spheroid area calculations. Occasionally an Incucyte image was found to be out of focus, with the out of focus image leading to a large discrepancy in values between adjacent time points. When this occurred, the unfocused image was excluded from the data set.

Drug potency determination in monolayers was performed by plating 3 × 10^3^ cells expressing red fluorescent nuclear protein on 96 well cell culture-treated plates. The cells adhered overnight prior to addition of drug and transfer to the live cell imager. As with the spheroids, the total red area signal was used to determine growth inhibition. A fixed threshold of 1.5 RCU was used to differentiate cells from background signal or debris.

### Quantitation of free drug

For each replicate and spheroid size, 12 spheroids were collected from individual wells and pooled together. The spheroids were centrifuged, and media supernatant was aspirated. The collection of spheroids was then washed once with phosphate-buffered saline, centrifuged, and supernatant aspirated. Cold organic solvent composed of 95:5 acetonitrile and methanol with 50 nM carbutamide was added to the spheroid pellet at a volume of 90 µL and vortexed. The mixture was centrifuged at 3500 RPM to pellet precipitated matter and the supernatant transferred to a plate containing both water and dimethyl sulfoxide (DMSO) for mass spectrometry analysis at a final composition of 18:2:1 organic solvent, water, and DMSO, respectively. A Sciex 5500 triple quadrupole mass spectrometer coupled to an Agilent 1290 liquid chromatography system with a CTC PAL autosampler was used for the liquid-chromatography tandem mass spectrometry (LC-MS/MS) analysis of free drug. A Kinetex 5 µm 100 A C18, 30 × 2.1 mm^2^ column was used with a 0.9 min LC gradient. Mobile Phase A was composed of HPLC grade water with 0.1% Formic Acid. Mobile Phase B consisted of HPLC grade acetonitrile with 0.1% Formic Acid. The selective reaction monitoring (SRM) transition used for cysteine-maleimidocaproyl-monomethyl auristatin F was set to 1046.6 *m/z* for the precursor mass and 428.1 *m/z* for the fragment mass. Standard curves were run alongside samples for conversion to drug concentrations.

### Data analysis

Values from live cell imaging experiments were imported into Prism 5 (GraphPad Software, La Jolla, CA). Least squares curve fits were obtained for potency calculations by inputting log-transformed data into the nonlinear regression function for log(agonist) versus response with variable slope. Statistical significance was calculated in Prism as well. All GR-related values (GR50 and Emax) were calculated using an online tool named GRcalculator^50^.

## Supplementary information

Supplementary information

Supplementary information2

Supplementary information3

Supplementary information4

Supplementary information5

Supplementary information6

## Data Availability

The datasets generated and/or analysed during the current study are available from the corresponding author upon reasonable request.
