# Supplementary material for: Effects of microtubule-inhibiting small molecule and antibody-drug conjugate treatment on differentially-sized A431 squamous carcinoma spheroids
Source: Sci Rep. 2020 Jan 22;10:907. doi: 10.1038/s41598-020-57789-y (PMC6976639; doi:10.1038/s41598-020-57789-y)
Supplement: Supplementary file 1 — Supplementary information [file 41598_2020_57789_MOESM1_ESM.pdf]

Effects of microtubule-inhibiting small molecule and antibody-drug conjugate treatment on differentially-sized A431 squamous carcinoma spheroids

Kenneth R. Durbin*, M. Shannon Nottoli, Gary J. Jenkins*

Drug Metabolism and Pharmacokinetics, AbbVie, Inc. North Chicago, IL 60064, United States.

*Corresponding authors: Kenneth R. Durbin, kdurbin@proteinaceous.net; Gary J. Jenkins, gary.jenkins@abbvie.com

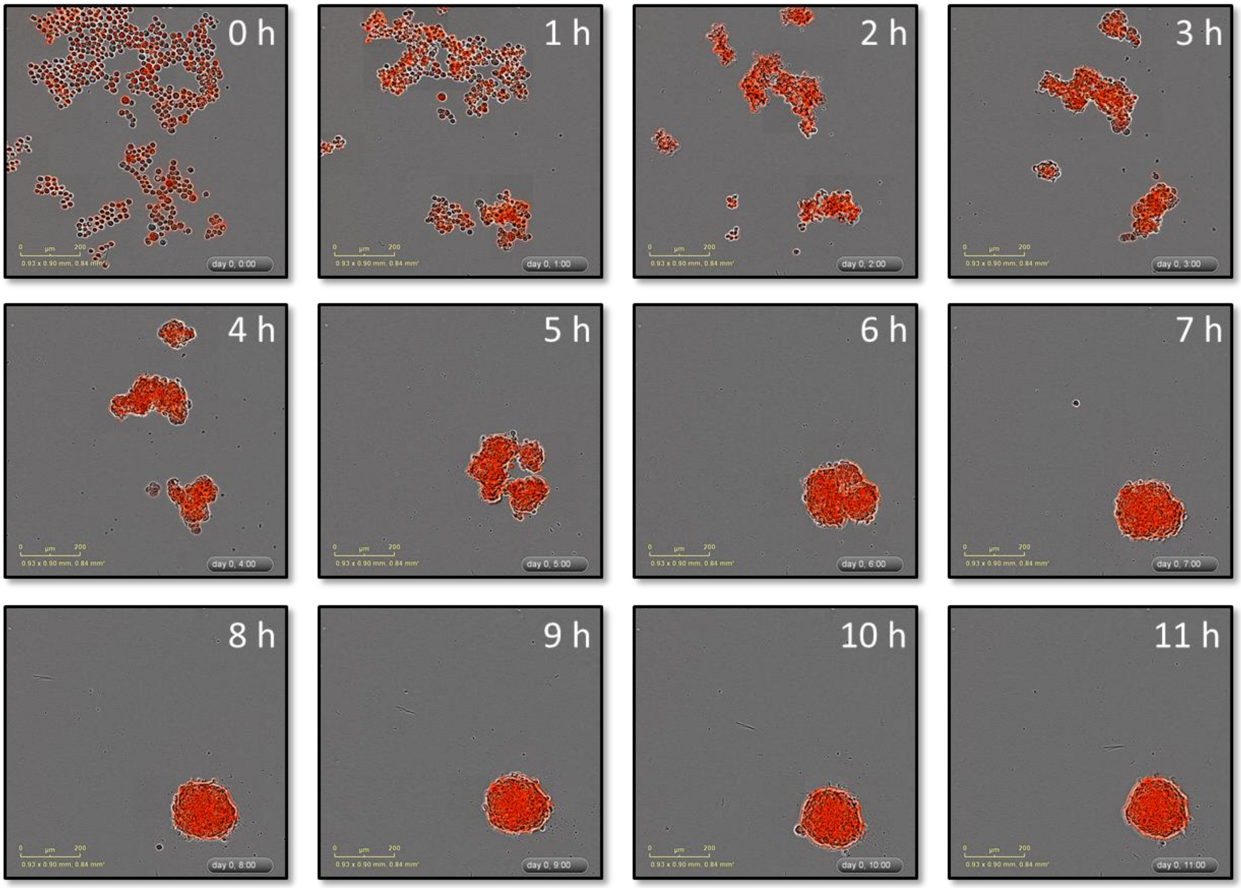

Supplemental Figure 1 – Spheroid formation. Fluorescently-labeled A431 cells were plated at 0.4×10^3 cells per well in an ultra-low adhesion plate. After centrifugation, the cells were observed using live cell imaging at one-hour intervals to monitor spheroid formation. Composite images of the phase and red channels are shown.

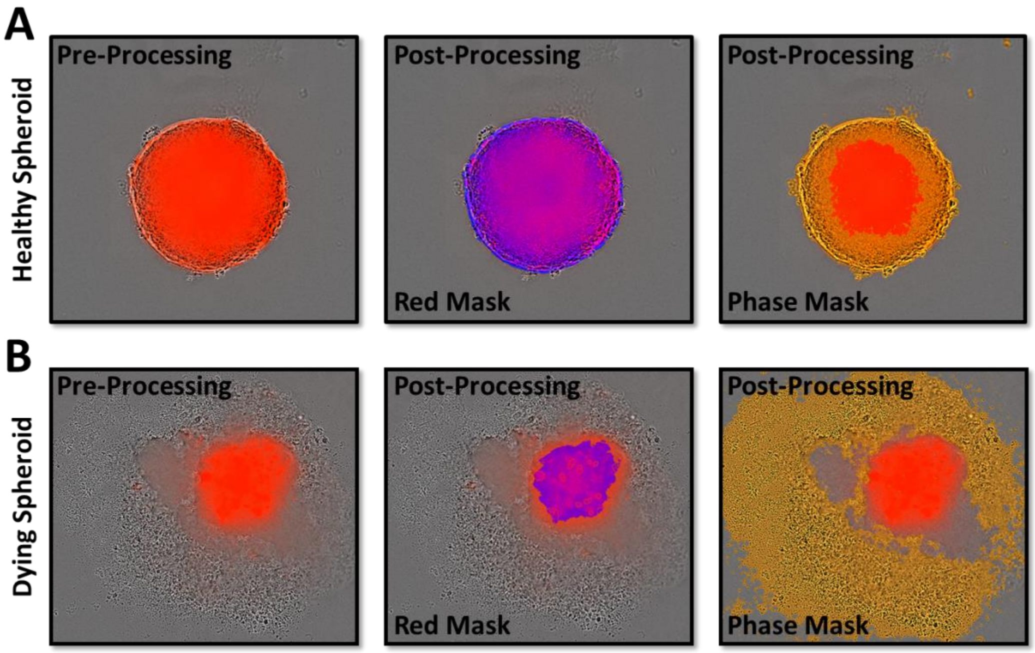

Supplemental Figure 2 – Determination of spheroid area. Data processing for live cell imaging of spheroids is shown for (**A**) a healthy spheroid and (**B**) a spheroid exhibiting significant cell death in the periphery. (**Left Panel**) Using live cell imaging, the total fluorescence signal from transfected nuclear proteins in A431 cells composing a spheroid could be tracked. (**Middle Panel**) The surface area was determined by using the area of fluorescent signal above a defined signal threshold to exclude any cells and debris not part of the spheroid, thereby only capturing the relevant spheroid portion. (**Right Panel**) Phase masking tends to produce inaccurate depictions of spheroid as shown with these two examples where the yellow mask, representing the spheroid region selected from the phase image, tends to include non-spheroid objects of the image as well as exclude portions of the spheroid.

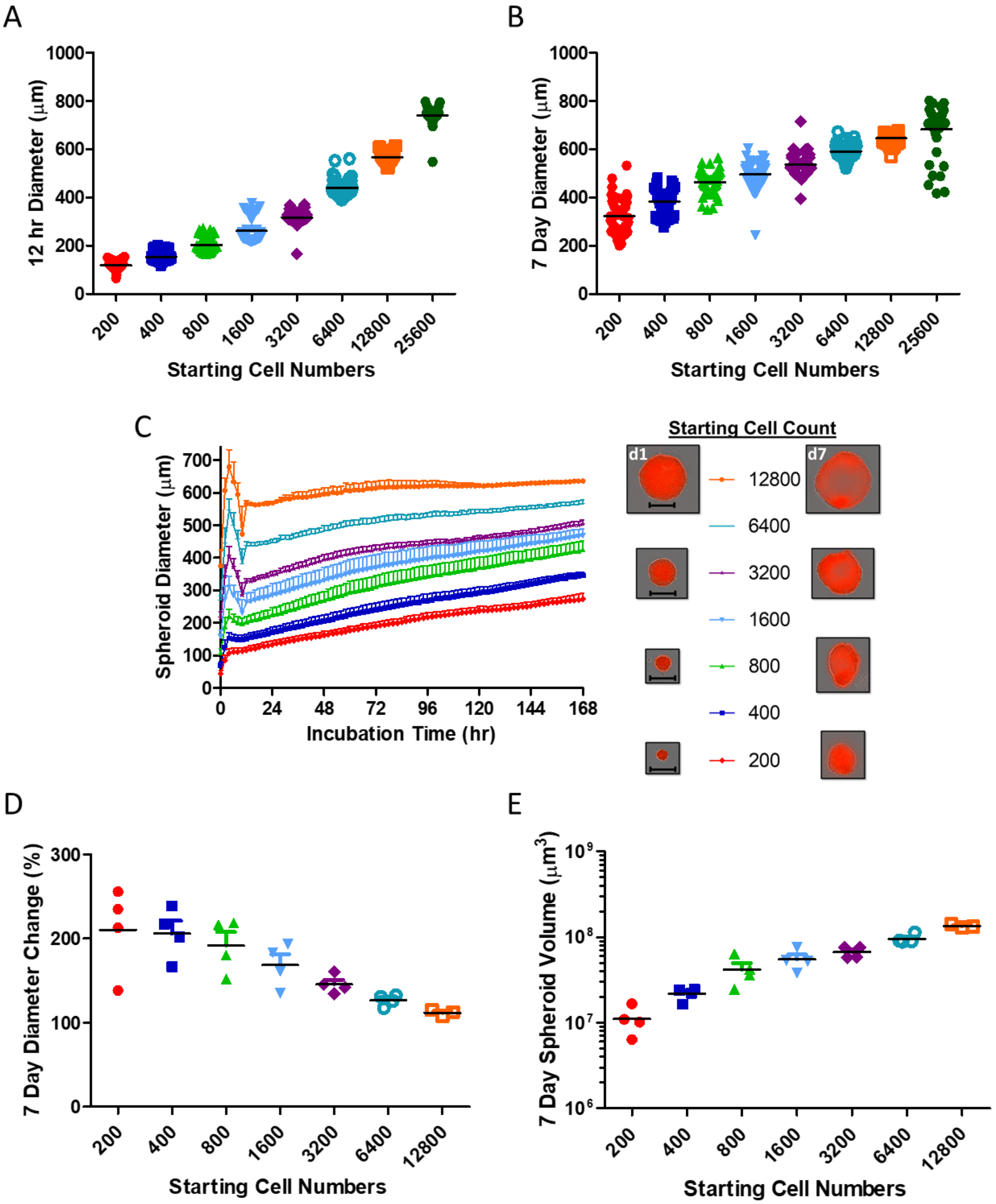

Supplemental Figure 3 – Spheroid diameter metrics. Diameters were calculated for all spheroids at each different starting cell seeding concentration after (A) 12 hr and (B) 7 days post-plating. Each concentration group contains ≥48 spheroids. (C) Spheroid diameter was tracked over 7 d by live cell imaging. Error bars are standard error of the mean from ≥3 separate experiments with each replicate experimental value the mean of ≥11 spheroids. From top to bottom, the images on the left side of the legend are day 1 spheroid images from 12800, 3200, 800, and 200 cell spheroids, respectively. The images on the right side are from the same spheroids on day 7. All spheroid images are on the same scale and the scale bars represent 300 μm. (D) The change in diameter for spheroids was calculated from the diameter at 12 hr post-plating (where

spheroids were fully formed) to the diameter at 7 d. (E) The spheroid volume was calculated from the diameter for all spheroids at 7 d post-seeding. The individual data points in D and E represent the mean value from one biological experiment while the line is the mean value from all replicates.

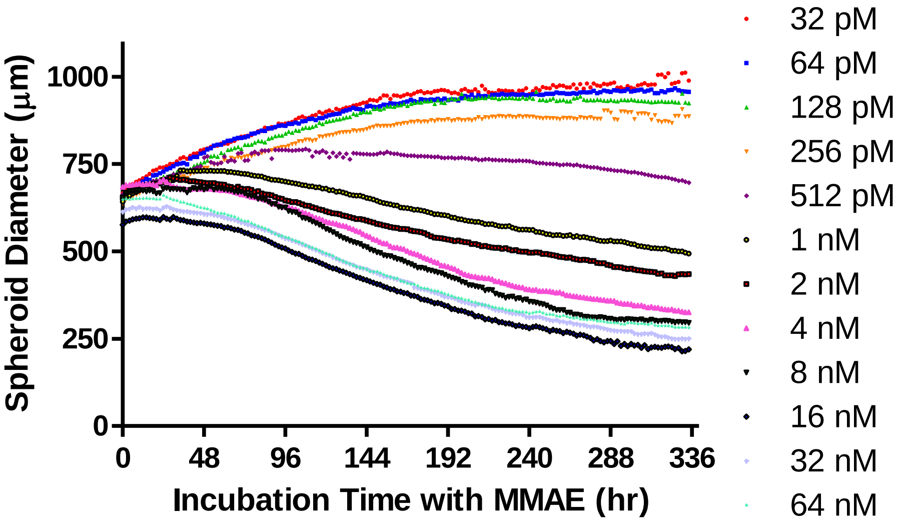

Supplemental Figure 4 – Spheroid treatment with MMAE. Spheroids were originally plated at 800 cells and allowed to grow for 9 days prior to treatment. Day 0 represents the start of treatment with MMAE following the initial 9-day growth period. Spheroid growth and reduction were tracked over 14 days while in the presence of MMAE. Data points are mean values from three biological replicates.

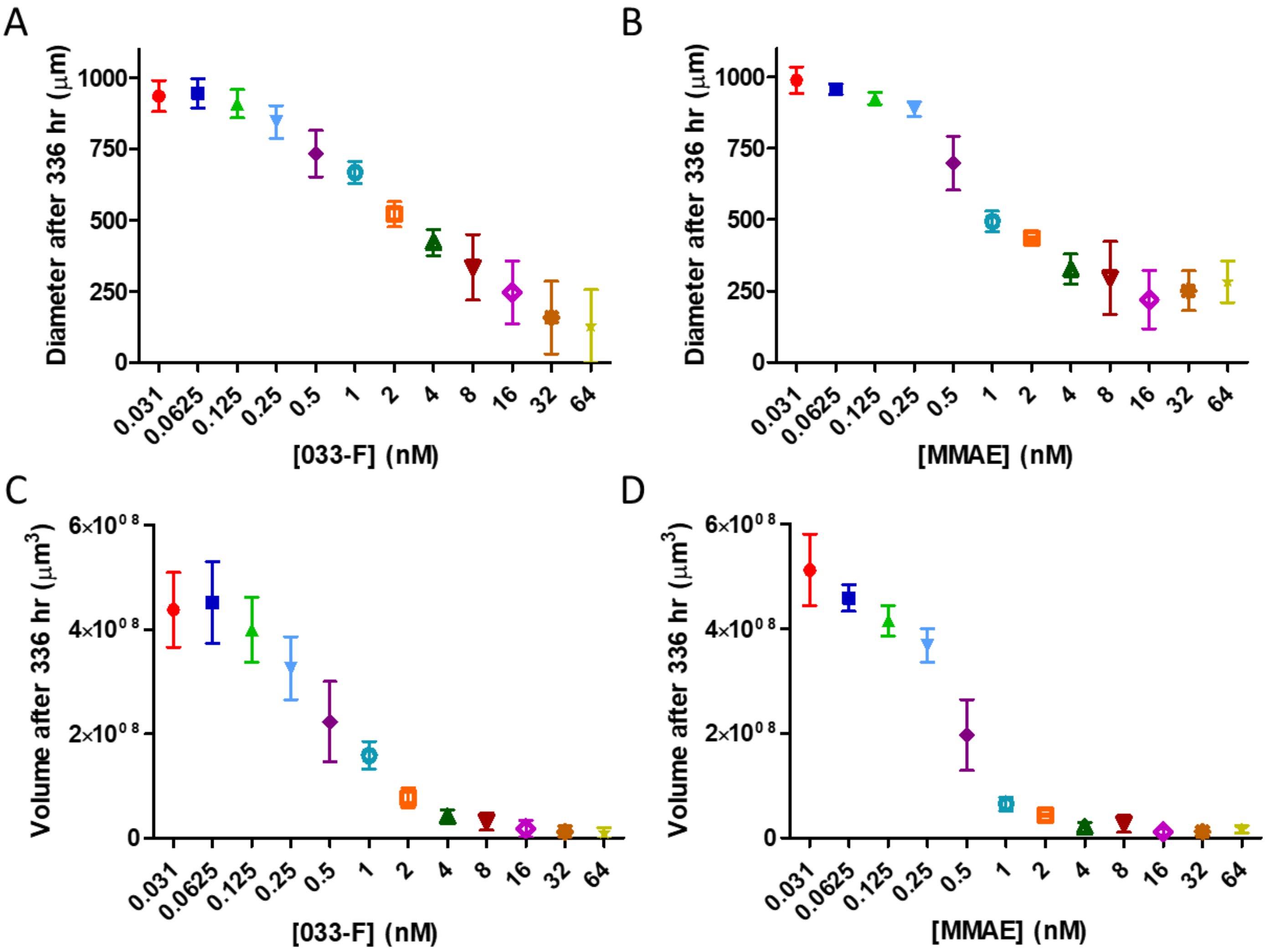

Supplemental Figure 5 – Spheroid metrics after treatment. Diameters were calculated for spheroids originally plated at 800 cells, grown for 9 days, and then treated for 14 days with (A) the 033-F ADC and (B) MMAE small molecule. The spheroid volumes were also calculated after 14 days of treatment with (C) 033-F and (D) MMAE. The mean values are shown from three biological replicates with error bars using standard deviation.

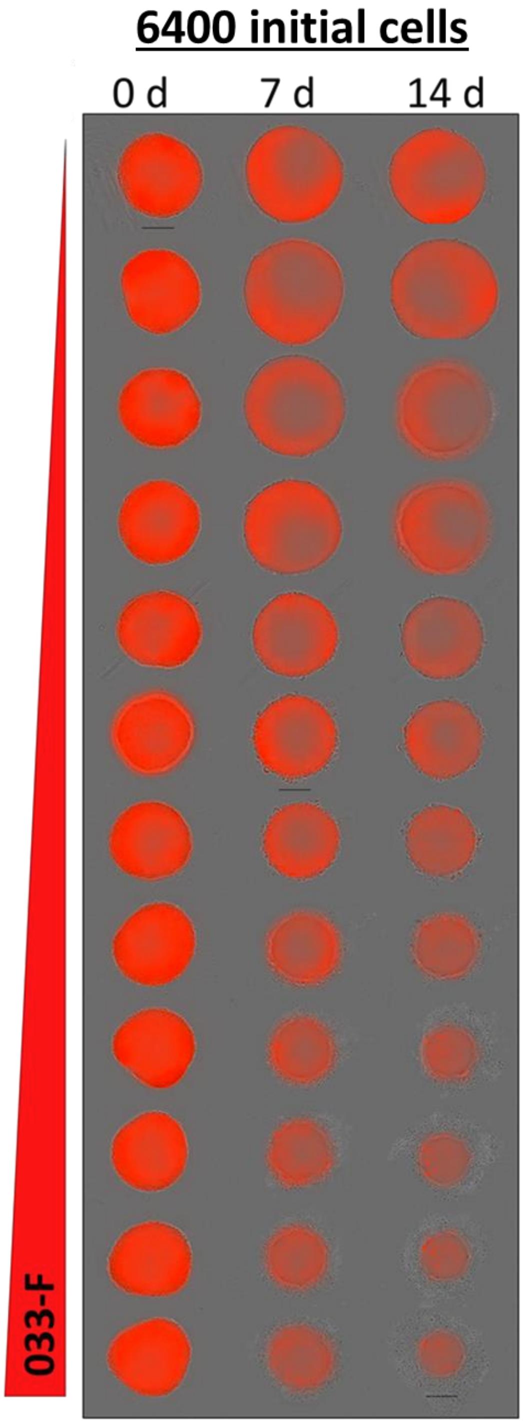

Supplemental Figure 6 – Treatment of spheroids with 033-F. Spheroids were originated from 6400 cells and treated with concentrations of 033-F varied by two-fold between 64 nM and 32 pM. Images from all concentrations of one experimental replicate are shown after 0, 7, and 14 d with 033-F.

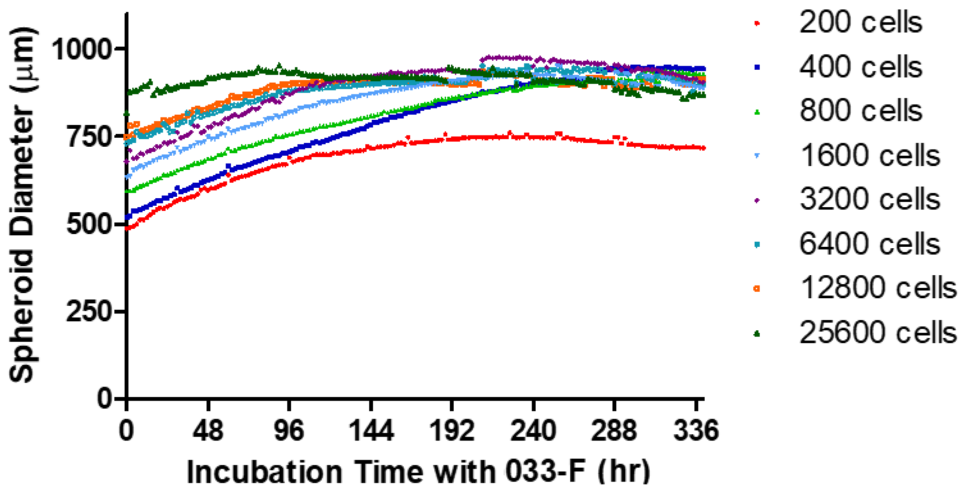

Supplemental Figure 7 – Spheroid growth at low 033-F concentrations. Spheroid diameters were tracked by live cell imaging for the different sizes of spheroids while being treated with 31 pM 033-F over 14 days.
